# Early life social defeat in mice reduces prefrontal innervation of downstream structures in a regionally specific manner

**DOI:** 10.1038/s41398-026-04229-6

**Published:** 2026-07-02

**Authors:** Miranda Mayeaux, Sunethra Shantha-Raman, Erin E. Hisey

**Affiliations:** 1 Program in Behavioral Neuroscience; Boston University Medical Center, Boston, MA 02118 USA; 2 VA Boston, West Roxbury, MA 02132 USA; 3 Boston University Chobanian and Avedesian School of Medicine, Boston, MA 02118 USA; 4 Department of Psychiatry; Boston University Medical Center, Boston, MA 02118 USA

**Keywords:** Molecular neuroscience, Depression

## Abstract

Physical abuse in childhood and adolescence results in long lasting effects on mental and physical health in adulthood. The prefrontal cortex of adults exposed to abuse in childhood shows both functional and structural abnormalities in comparison to healthy controls. However, the changes in prefrontal innervation of *specific* downstream targets as a result of early life abuse are unclear. Here we used a novel abbreviated early adolescent chronic social defeat stress paradigm (5 day eaCSDS) to assess changes to prefrontal innervation in adult male and female C57BL/6 J mice. We injected control and defeated mice with virally encoded GFP in the prelimbic region of prefrontal cortex (PL) and examined axon density in basolateral amygdala (BLA), nucleus accumbens (NAc) and ventral tegmental area (VTA). We find that both male and female mice show robust but seemingly opposing changes in social interaction after 5 day eaCSDS. We found significantly decreased PL innervation of BLA and increased fragmentation of PL axons in BLA. Males, but not females, also show increased fragmentation of PL axons in NAc. Interestingly, PL innervation of BLA significantly correlates with adult social behavior in males and females. Our findings suggest that PL innervation of downstream targets is dramatically affected by early life social defeat in both a regional and sex-specific manner. This differential change in innervation across regions and sexes may underlie the social behavioral deficits seen after early life social defeat.

## Introduction

Physical abuse experienced in childhood and adolescence is a leading predictive factor of the development of psychiatric disease in adulthood [1, 2]. Complex PTSD [3] and personality disorders [4, 5], in particular, are thought to result from early life abuse in the majority of patients. These disorders are marked by difficulties in social interaction and in forming social relationships [6, 7]. It has long been suggested that early life abuse disrupts prefrontal control over downstream structures, “removing the brakes” over areas that may increase fear or anxiety in the absence of top down control [8–11]. Indeed, functional imaging studies have shown decreased prefrontal cortical (PFC) activation during emotional regulation and symptom provocation tasks in patients with PTSD and childhood trauma [12–18]. This decrease in prefrontal activation is often accompanied by an increase in reactivity in downstream structures such as the amygdala [19–21]. While diffusion tensor imaging evidence in humans suggests that the structure of white matter within the PFC [22–24] as well as the structure of the cingulum, the axon bundle connecting the prefrontal cortex to downstream regions [25], is altered after trauma, it is unclear how PFC innervation of specific downstream targets is affected.

Here we examined prefrontal innervation of downstream structures including basolateral amygdala (BLA), nucleus accumbens (NAc), and ventral tegmental area (VTA) anatomically using a mouse model of early life physical abuse. Early adolescent chronic social defeat stress (eaCSDS), in which a young (~P30) mouse is subjected to repeated aggressive physical encounters followed by housing beside the aggressor, has been shown to dramatically alter social interaction in adulthood in males [26]. The prelimbic cortex has been shown to be critical for social interaction after defeat in adult male mice [27] but its role in social interaction after early life defeat is unknown. While it is known that early life abuse disrupts white matter integrity in PFC of humans, it is unknown whether anatomical connectivity between PL and key downstream regions is disrupted in mice subjected to protocols designed to approximate abuse. Using fluorescent tracer injections in adult mice subjected to an abbreviated eaCSDS protocol, we examine PL innervation of downstream regions in a target- and sex-specific manner. We then performed correlational analyses between social behavior in defeated animals and changes in PL innervation.

## Methods

### Subjects

3 week old male and female C57BL/6J (C57) mice were either obtained from Jackson Laboratory (JAX, Bar Harbor, ME, USA) or bred in the VA Boston-West Roxbury animal facility. JAX mice were shipped at P21+/− 3 days. The mice were maintained on a 12 h light, 12 h dark schedule and provided food and water ad libitum. All experimental procedures described below were approved by the VA Boston Institutional Animal Care and Use Committee and were performed in accordance with the National Institutes of Health’s Guide for the Care and Use of Animals. Sample size was determined by previous size of cohorts used in the Hisey lab to reach significance. Mice were assigned to control and defeated cohorts randomly and average weights of animals for each group were compared before the start of defeat to ensure no statistical difference in starting weight.

### 5 day early adolescent chronic social defeat stress (eaCSDS) in C57 mice

#### Generation of aggressors

Virgin male and female CFW mice (8 weeks) were obtained from Charles River (Boston, MA, USA). Males were housed with ovariectomized female CFWs (10 weeks, Charles River) while females were housed with castrated male CFWs (10 weeks, Charles River) for at least 1 week to induce territorial aggression. Males were screened for aggression against C57 adolescent males while females were screened for aggression against C57 adolescent females. Only aggressor males and females with attack latencies of less than 30 s for 2 consecutive days were used in eaCSDS.

#### 5 day eaCSDS

On P30, male and female early adolescent C57s mice were subjected to an aggressive encounter (30 bites or 3 min, whichever occurred first) with a sexually experienced adult CFW mouse of the same sex as described previously [26]. After the aggressive encounter, a plexiglass partition was placed between the mice that allowed for sensory but not physical contact. They were then housed overnight until the following day when the early adolescent mice were defeated and then housed beside a new aggressor. Previously, we used a 10 day defeat period [26]; however, we wanted to determine if similar effects on sociability could be achieved in a shorter time period more akin to the duration of mouse puberty. Thus, we proceeded with defeats for a total of 5 days, after which the defeated mice were housed beside each other with a plexiglass partition dividing the cage. To control for potential effects of social isolation, male and female early adolescent mice were housed side-by-side, separated by a plexiglass partition, from P30 onward.

### Social behavior testing

#### Open field social interaction (OFSI)

eaCSDS was performed as described previously^26^. Briefly, control and defeated C57 mice were moved from the housing room into the behavioral testing room and habituated to the room for one hour. Each mouse was then placed in a 18”x18” x18” square opaque acrylic arena with a wire cup placed against the wall for 150 s. The mouse was then removed from the arena and a nonaggressive CFW mouse of the same sex was placed inside the wire cup. The C57 mouse was then reintroduced to the arena for 150 s. All behavior was recorded in Ethovision and the social interaction zone was labeled as the ~2 cm circular region surrounding the wire cup. Social interaction ratio was calculated as the amount of time spent in the social interaction zone when the CFW is inside the cup divided by the amount of time spent in the social interaction zone when the cup is empty.

#### Home cage social interaction (HCSI)

eaCSDS was performed as described previously^26^. Control and defeated C57 mice were moved from the housing room into the behavioral testing room and habituated to the room for one hour. Mice were then placed in a clean home cage for one minute. Then, an age matched C57 mouse of the same sex was placed in the arena for 90 s. All behavior was recorded in Ethovision and social behavior was assessed using the Social Interaction Module.

### Viral injections

Mice were anesthetized and mounted in a standard stereotaxic apparatus. The skull was exposed and coordinates relative to bregma, lambda, and midline were measured. A hole was drilled over the prelimbic region of the prefrontal cortex (PL, +2.2 mm A/P from Bregma, 0.3 mm M/L from Bregma, −1.2 mm D/V from the surface of the brain) for a unilateral injection and a glass pipette attached to a Nanoject pressure injection system was slowly lowered to the appropriate depth. The hemisphere injected was counterbalanced across cohorts. Pipettes were left in place for 5 min before and following injection to prevent viral efflux and facilitate diffusion before being slowly retracted. Each mouse was injected with 2 injections of 18 nl AAV9-hSyn-GFP (Addgene) with one minute between injections. The incision site was then sutured and the mouse removed from the stereotaxic apparatus and placed in their homecage atop a heating pad. Mice were then injected with meloxicam (5 mg/kg) subcutaneously to reduce inflammation from surgery. After mice regained sternal recumbency, they were returned to their housing room and monitored daily for the next 4 days for signs of pain or infection. 24 h after surgery, they were again injected with meloxicam (5 mg/kg) intraperitoneally.

### Histology

Mice were deeply anesthetized with isofluorane then transcardially perfused with 20 ml of phosphate buffered saline (PBS) followed by 20 ml of 10% formalin. Brains were removed and incubated in 10% formalin for 24 h then moved to a 30% sucrose-PBS solution for another 24 h. Brains were then embedded in OCT tissue cutting media and continuously sectioned at 50 um. Alternate sections were mounted and allowed to dry overnight before coverslipping with DAPI-containing mounting medium (VECTASHIELD HardSet with DAPI).

### Microscopy and image analysis

Brain tissue sections were imaged using a Zeiss Axioskop. Images were acquired using Stereoinvestigator. At least 6 sections of each region of interest (PL, basolateral amygdala (BLA), nucleus accumbens (NAc), ventral tegmental area (VTA)) from each mouse were imaged. PL images containing the injection site were taken at 2.5x magnification. BLA, NAc, and VTA images were taken at 5x.

All image analysis was performed in ImageJ/FIJI by an experimenter blinded to experimental condition. Integrated density was measured from a 300 um^2^ square region of interest in BLA, NAc, and VTA, centered on the area containing the most dense PL axonal innervation using the Measure function. Integrated density is defined as the product of the area labeled and the mean intensity. While percent area is commonly reported [28, 29], we chose to use integrated density to better capture the intensity of labeling. The integrated density of background fluorescence was measured from a square region of tissue of equivalent size outside the region of interest and subtracted from the integrated density for the region of interest; this region was kept consistent across mice for each region [30]. For the BLA, this was the region directly lateral of the BLA ROI that did not contain fluorescent axonal labeling. For the NAc, this was the region directly lateral to the NAc ROI that did not contain fluorescent axonal labeling. For the VTA, this was the region that did not contain fluorescent axon labeling and was immediately above the VTA ROI. Particle analysis was used to measure the size and fragmentation of thresholded axons within a larger region of interest (800 × 1000 um^2^ for BLA and NAc, 400×650 um^2^ for VTA). Particle size and number of particles were obtained using the Threshold function Otsu filter then the Analyze Particles function (default settings) as described previously^32^. Percent area and mean fluorescent intensity were obtained using the Threshold function Default filter then the Measure function.

### Statistical analysis

All behavioral and histological data were analyzed in GraphPad Prism 10. The data were first tested for outliers using the ROUT outlier test (p > 0.05). Outliers were then removed from statistical analysis and noted in figure legends. The data was then tested for normality using the Shapiro-Wilk normality test. If data passed the normality test (p > 0.05), parametric analyses were used; if the normality test failed, non-parametric analyses were used. Differences in variance across compared groups was calculated and statistical tests were chosen based on whether variances were or were not statistically comparable.

## Results

**Fig. 1:**
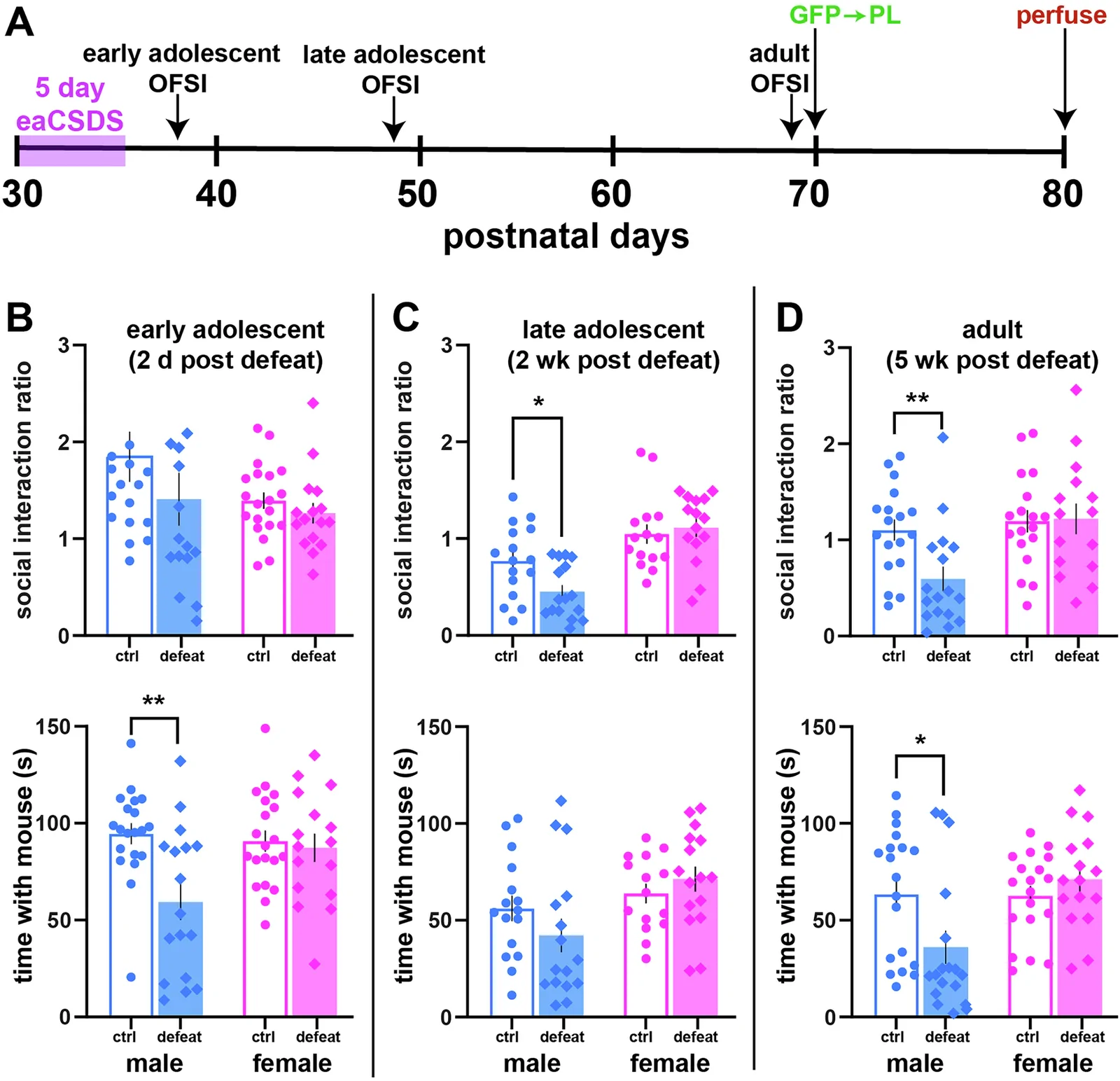
5 day eaCSDS impairs social interaction in OFSI in males but not females. **A** Schematic of experimental design. **B** Social interaction ratio and time spent in interaction zone with CFW present 2 days after defeat (n = 18 control males, 17 defeated males, 18 control females, 15 defeated females) (time with mouse (males): Mann Whitney test, p = 0.0039, U = 77). **C** Social interaction ratio and time spent in interaction zone with CFW present 2 weeks after defeat (social interaction ratio (males): Mann Whitney test, p = 0.0139, U = 63.50). **D** Social interaction ratio and time spent in interaction zone with CFW present 5 weeks after defeat (social interaction ratio (males): Mann Whitney test, p = 0.0026, U = 64; time with mouse (males): Mann Whitney test, p = 0.0194, U = 88).

We first wanted to establish if a 5 day defeat protocol in a peripubescent time window would produce robust deficits in social behavior as seen with the longer 10 day protocol. On P30, male and female early adolescent C57BL/6 J mice were subjected to an aggressive encounter with a sexually experienced adult CFW mouse of the same sex. They were then housed overnight separated from one another with a plexiglass barrier until the following day when the early adolescent mice were defeated by and then housed beside a new aggressor. This process continued for a total of 5 days, after which the defeated mice were housed beside each other with a plexiglass partition dividing the cage. To control for potential effects of social isolation, control male and female early adolescent mice were housed side-by-side, separated by a plexiglass partition from P30 onward (Fig. 1A).

**Fig. 2:**
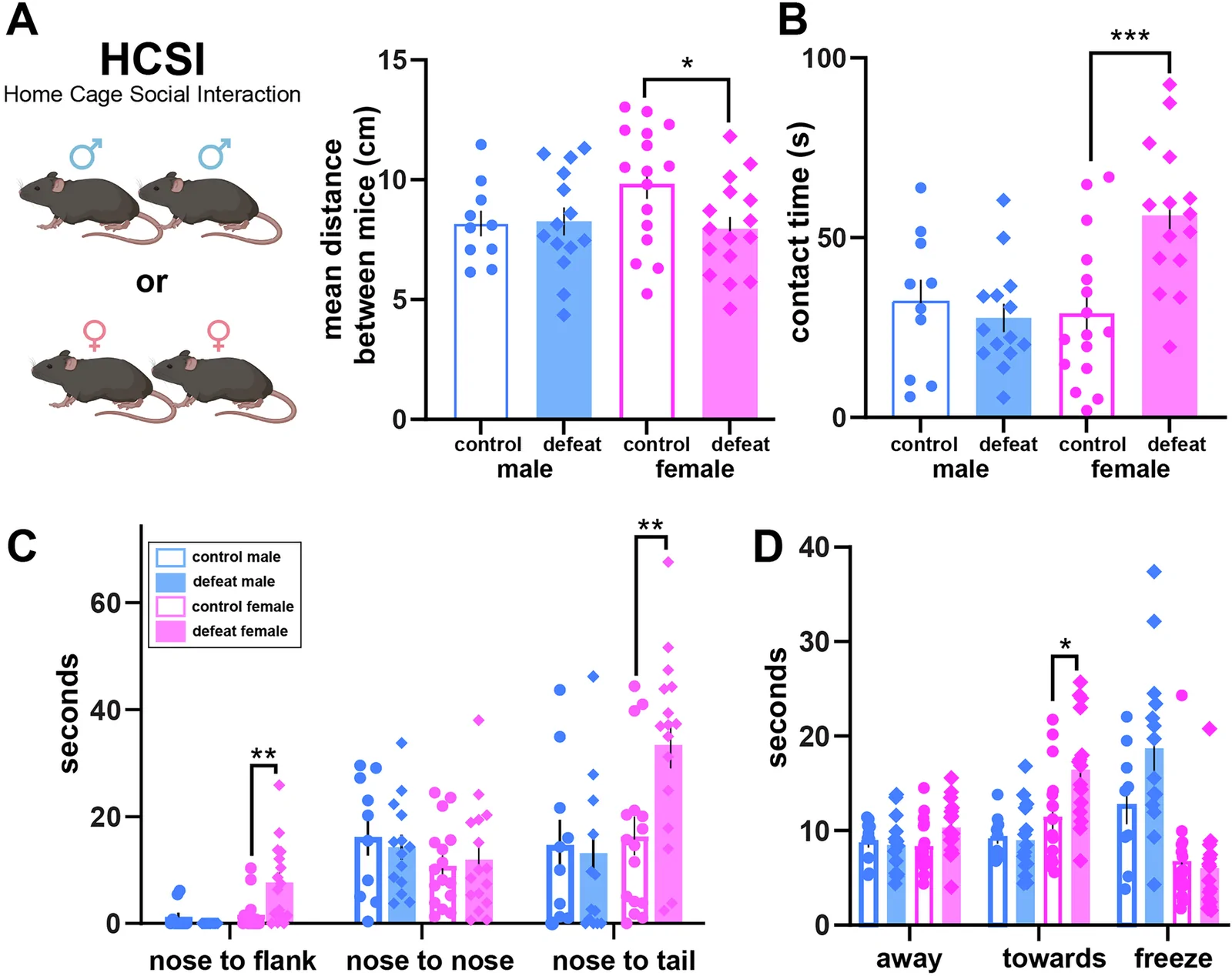
5 day eaCSDS impacts female, but not male, social behavior in same-strain free social interaction. **A** Average distance between mice during 90 s of home cage social interaction (n = 10 control males, 13 defeated males, 16 control females, 16 defeated females) (unpaired two-tailed t test in females, p = 0.0251, F = 1.582). **B** Contact time between mice (unpaired two-tailed t test in females, p = 0.0008, F = 1.008). **C** Time spent engaging in social behaviors (nose to flank (of stimulus mouse), unpaired two-tailed t test in females, p = 0.0061, F = 5.797; nose to tail (of stimulus mouse), unpaired two-tailed t test in females, p = 0.0053, F = 1.431). **D** Time spent in movement state (movement towards stimulus mouse, unpaired two-tailed t test in females, p = 0.0131, F = 1.161).

We assessed social behavior using open field social interaction at 3 different timepoints (2 days after, 2 weeks after, 5 weeks after) as described previously to gauge behavioral effects of the 5 day defeat (Fig. 1B–D). In males, we saw reduced social interaction ratios in late adolescence and adulthood, as well as reduced social interaction time with the CFW stimulus mouse in early adolescence and adulthood (Fig. 1B–D). Interestingly, in females we saw no significant differences between defeated and control animals in either social interaction ratio or social interaction time at any time point (Fig. 1B–D). We then wanted to determine if defeat affected social interaction with a freely behaving mouse of the same sex, strain and age. We used home cage social interaction (HCSI), which allows for free interaction between mice, to assay specific behavioral changes in both male and female mice. Interestingly, we saw marked social interaction changes in defeated females but not males. In females we saw significantly decreased distance from the stimulus mouse (Fig. 2A) and significantly increased time in contact with the stimulus mouse (Fig. 2B), specifically in terms of increased nose to flank and nose to tail contact (Fig. 2C). We also noted significantly increased amount of time spent approaching the stimulus mouse (‘movement towards’) in females (Fig. 2D). Surprisingly we saw no significant changes in any of the quantified social behavior in defeated males compared to controls. Overall, we were able to recapitulate the hallmark outcome of our 10 day eaCSDS protocol (social avoidance in OFSI in males) with 5 day eaCSDS as well as extend the use of eaCSDS to females. Interestingly, we saw no effects in OFSI in defeated females but found aberrant pro-approach behaviors in defeated females during interaction with an age, strain, and sex matched mouse.

**Fig. 3:**
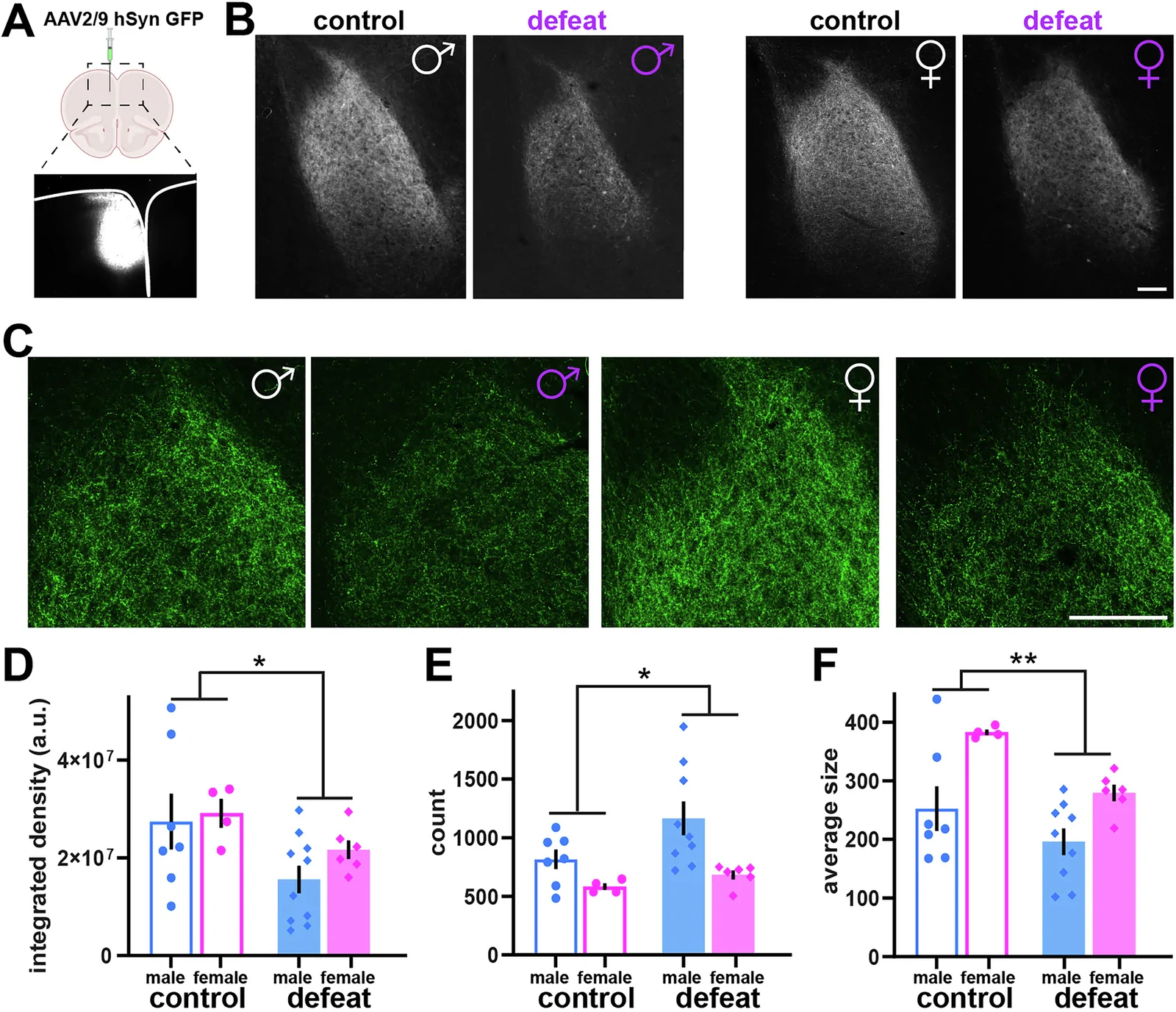
PL axonal innervation of BLA is decreased and fragmented after eaCSDS in both males and females. **A** Schematic of injection and example image of injection site. **B** Images of BLA at 5x magnification. Scale bar, 200 um. Purple symbols denote defeated animals, white symbols denote control animals. **C** Images of BLA at 20x magnification. Scale bar, 200 um. **D** Integrated density of PL axons in BLA (males: n = 7 control animals, 10 defeated animals; females: n = 4 control animals, 6 defeated animals) (main effect of defeat, 2 way ANOVA, F(1,23) = 6.799, p = 0.0157, 95% CI: control: [1.99E7, 3.62E7], defeated: [1.34E7, 2.20E7], Cohen’s f = 0.544). **E** Number (count) of fluorescently labeled particles in BLA (males: n = 7 control animals, 9 defeated animals (1 animal removed due to inability to image entire ROI (800 × 1000 um^2^) without tears in tissue); females: n = 4 control animals, 6 defeated animals) (main effect of defeat, 2 way ANOVA, F(1,22) = 4.381, p = 0.0481, 95% CI: control: [588.6, 869.6], defeated: [747.9, 1205], Cohen’s f = 0.446; main effect of sex, 2 way ANOVA, F(1,22) = 10.85, p = 0.0033, 95% CI: males: [809.3, 1224], females: [573.4, 708.0], Cohen’s f = 0.702). **F** Average size of fluorescently labeled particles in BLA (males: n = 7 control animals, 9 defeated animals; females: n = 4 control animals, 6 defeated animals) (main effect of defeat, 2 way ANOVA, F(1,22) = 9.605, p = 0.0052, 95% CI: control: [231.6, 368.7], defeat: [189.3 267.1,], Cohen’s f = 0.661; main effect of sex, 2 way ANOVA, F(1,22) = 17.01, p = 0.0004, 95% CI: males: [173.8, 265.6], females: [278.4, 363.5], Cohen’s f = 0.879).

We then wanted to determine if 5 day eaCSDS could result in anatomical impacts on the adult brain. To examine changes in prefrontal axonal innervation of downstream targets, we injected an AAV encoding GFP unilaterally into the prelimbic cortex (PL) of control and defeated mice at P70 (Fig. 3A). We sacrificed the mice 10 days later and serially sectioned the brains, mounting every other section (see Fig. 1A for experimental timeline). We examined the PL injection site to assess the spread of the injection and only included animals with injections that spread throughout PL (no less than 1.5 x 10^5^ um^2^ average area observed across at least 5 sections, Supplemental Fig. 1A) without spillage into infralimbic cortex in our analysis.

To further ensure the observed changes in axonal density were not confounded by potential variability in injection site labeling density or spread, we compared injection site integrated density (averaged across 5 sections) between control and defeated animals and found no significant difference (Supplemental Fig. 1B). We further correlated integrated density of fluorescently labeled PL axons in BLA with average injection site area per animal (Supplemental Fig. 1C). We found no significant correlation between PL axons in BLA and average injection site area in either male or female cohorts, suggesting that the changes observed in axonal innervation are unlikely to be due to variability in injection size.

We then examined innervation of the basolateral amygdala (BLA), the nucleus accumbens (NAc), and the ventral tegmental area (VTA) as these are primary downstream targets of PL found to be involved in the modulation of social behavior [31–33]. To measure the total fluorescent signal within a region, we used ImageJ to calculate the integrated density, or the area labeled multiplied by the fluorescent intensity, within a 300 um^2^ box centered on the anatomical region of interest from which we subtracted the integrated density from an equivalently sized region directly lateral to the site of interest but without fluorescent label [30]. Additionally, we calculated mean fluorescent intensity and percent area within the same 300 um^2^ box centered on the ROI with a default filter applied to automatically remove background signal. To measure the composition and size of the labeled axonal field on a larger scale, we again used ImageJ to calculate the number and size of detected fluorescent particles within a rectangle (800 × 1000 um^2^ for BLA and NAc, 400x650 um^2^ for VTA) centered on the anatomical region of interest [34].

In BLA we found significantly reduced integrated density of fluorescently labeled PL axons in both male and female defeated mice (main effect of defeat, 2 way ANOVA, p = 0.0157; Fig. 3B–D). We also found reduced percent area in male defeated mice (interaction between sex and defeat, 2 way ANOVA, p = 0.0360, Tukey HSD for control vs defeated males, p = 0.0288; Supplemental Fig. 2A) and reduced mean fluorescent intensity in both male and female defeated mice (main effect of defeat, 2 way ANOVA, p = 0.0455; Supplemental Fig. 2B). We found an increased number of labeled particles, a phenotype consistent with axonal fragmentation and degeneration [34–36], in both defeated males and females (main effect of defeat, 2 way ANOVA, p = 0.0481; Fig. 3E). We additionally found reduced size of labeled particles, a phenotype also consistent with axonal fragmentation and degeneration [34–36], in both defeated males and females (main effect of defeat, 2 way ANOVA, p = 0.0052; Fig. 3F). We found a main effect of sex in both particle count (main effect of sex, 2 way ANOVA, p = 0.0033) and particle size (main effect of sex, 2 way ANOVA, p = 0.0033). In summary, in both males and females we saw indications of decreased PL innervation of BLA as well as indications of fragmentation with increased particle count and decreased particle size. Please see Supplementary table 1 for additional statistical information for the ANOVAs described above.

**Fig. 4:**
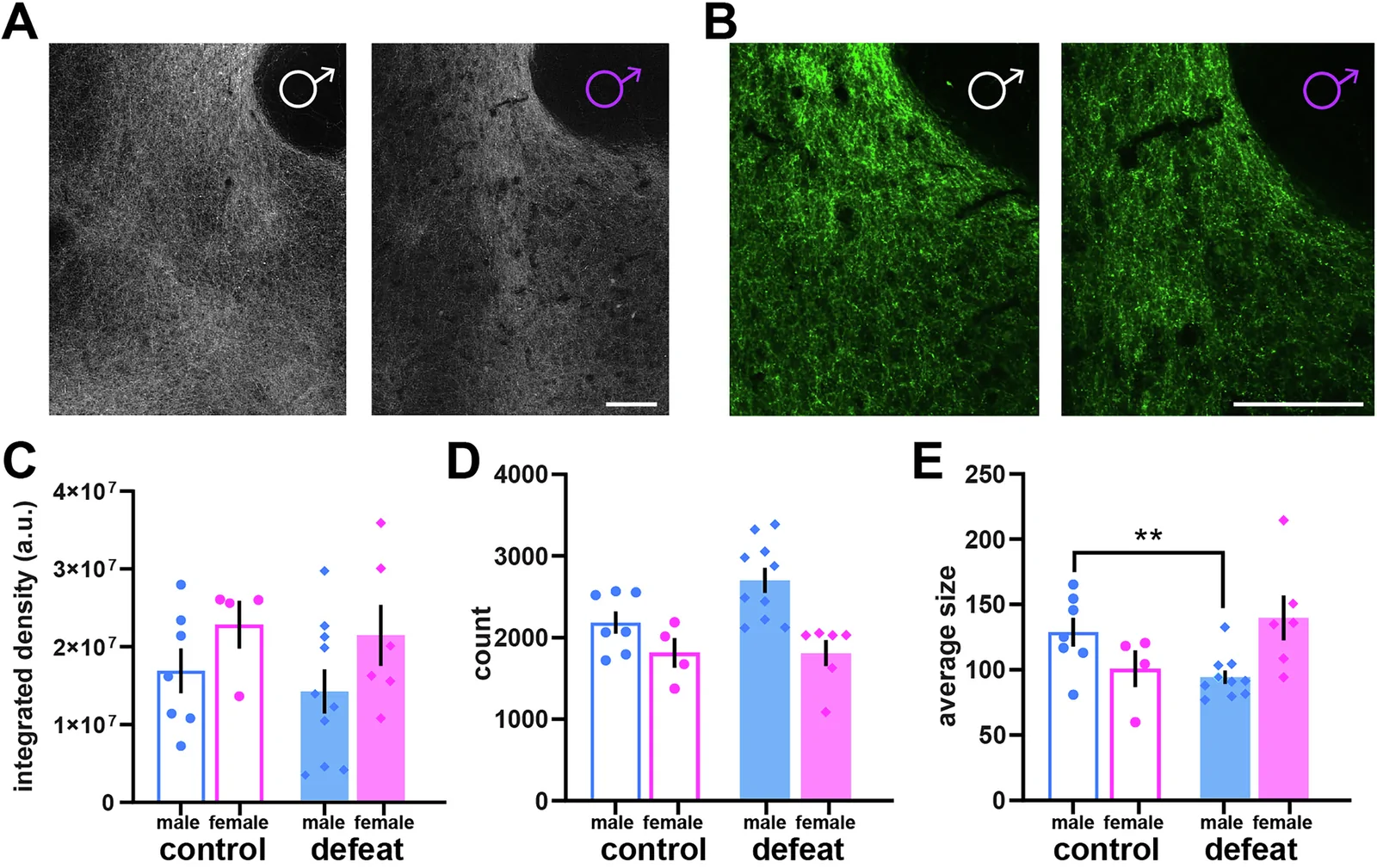
PL axonal innervation of NAc is decreased in males and females and fragmented only in males after eaCSDS. **A** Images of NAc in control and defeated males at 5x. Scale bar, 200 um. Purple symbols denote defeated animals, white symbols denote control animals. **B** Images of NAc in control and defeated males at 20x. Scale bar, 200 um. **C** Integrated density of PL axons in NAc (males: n = 7 control animals, 10 defeated animals; females: n = 4 control animals, 6 defeated animals) (main effect of sex, 2 way ANOVA, F(1,23) = 4.427, p = 0.0465, 95% CI: males: [1.09E7, 1.95E7], females: [1.63E7, 2.77E7], Cohen’s f = 0.439). **D** Number (count) of fluorescently labeled particles in NAc (main effect of sex, 2 way ANOVA, F(1,23) = 15.42, p = 0.0007, 95% CI: males: [2223, 2733], females: [1555, 2067], Cohen’s f = 0.819). **E** Average size of fluorescently labeled particles in NAc (interaction between sex and defeat, 2 way ANOVA, F(1,23) = 12.67, p = 0.0017, 95% CI: control males: [102.10, 155.40], defeated males: [81.05, 104.30,], control females: [55.98, 145.60,], defeated females: [95.78, 183.8], Cohen’s f = 0.742; Tukey HSD, p = 0.0048).

In NAc (Fig. 4A, B), we did not find any effects of defeat on either PL axon integrated density, mean fluorescent intensity, or particle count but did find a main effect of sex for these three measures (main effect of sex for integrated density, 2 way ANOVA, p = 0.0465; main effect of sex for mean intensity, 2 way ANOVA, p = 0.0202; main effect of sex for particle count, 2 way ANOVA, p = 0.007) (Fig. 4C, D, Supplemental Fig. 2D). We found no interaction or main effects of sex or defeat on percent area (Supplemental Fig. 2C). We found a significant decrease in average particle size in defeated males, but not defeated females (interaction of sex and defeat, 2 way ANOVA, p = 0.0017; Tukey HSD, p = 0.0048) (Fig. 4E). Please see Supplementary table 1 for additional statistical information for the ANOVAs described above.

When we examined PL innervation of VTA we found no interaction effects or main effects of either sex or defeat on integrated density, percent area, particle count, or average particle size (Supplemental Fig. 3, Supplementary table 1) though we did find a significant effect of sex for mean fluorescent intensity (main effect of sex for mean intensity, 2 way ANOVA, p = 0.0375). To summarize, we found the most robust decreases in measures of PL axon density and increased PL axon fragmentation in the BLA of both males and females. Defeated males also showed a significant decrease in average particle size in NAc. Females showed no significant effects of defeat in any measure of innervation in either NAc or VTA. Interestingly, we found a significant main effect of sex on PL innervation in both BLA (particle count, particle size) and NAc (integrated density, mean intensity, particle count, particle size).

**Fig. 5:**
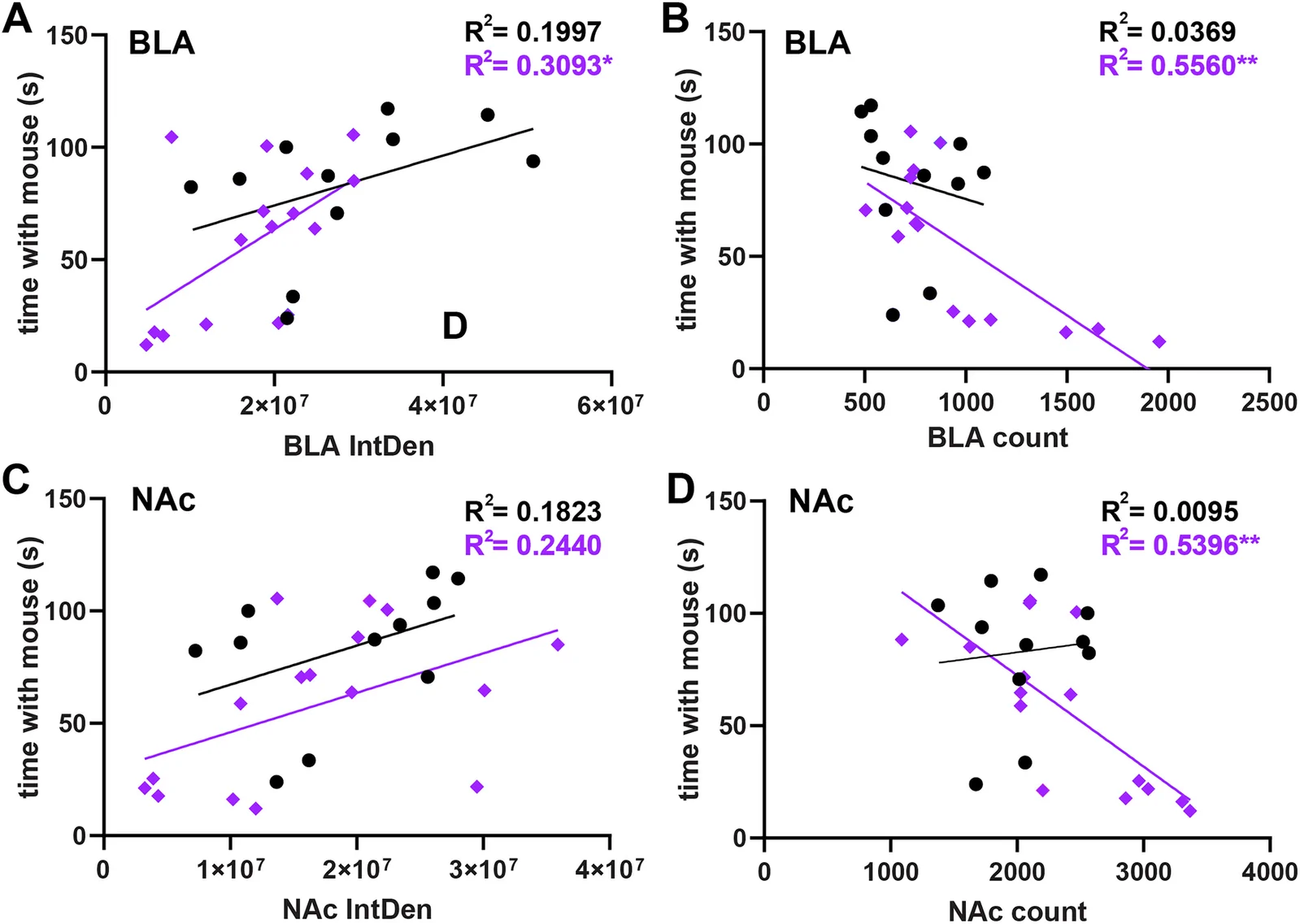
Correlations between PL axonal innervation of downstream regions and social interaction. Time spent in interaction zone with CFW present 5 weeks after defeat for all males and females (black, n = 11 controls; purple, n = 16 defeated) as a function of **A**) integrated density of PL axons in BLA (defeated: simple linear regression, R^2^ = 0.3093, F(1,14) = 6.270, p = 0.0253) or **B**) number (count) of fluorescently labeled particles in BLA (defeated: simple linear regression, R^2^ = 0.5560, F(1,13) = 16.28, p = 0.0014). Time spent in interaction zone with CFW present 5 weeks after defeat for all males and females as a function of **C**) integrated density of PL axons in NAc or **D**) number (count) of fluorescently labeled particles in NAc (defeated: simple linear regression, R^2^ = 0.5396, F(1,14) = 16.41, p = 0.0012).

Given the range of variability in PL innervation of BLA in both males and females, we wanted to determine if there was a relationship between social behavior and loss of innervation. We examined the correlation between the amount of time spent with the CFW mouse during OFSI 5 weeks after defeat in both control and defeat groups (groups were collapsed by sex to include all males and females) and PL innervation density and count in BLA. We found a significant positive correlation between the time spent in the interaction zone with a CFW present and the integrated density of PL axons in BLA (Fig. 5A, R^2^ = 0.3093, p = 0.0253). We also found a significant negative correlation between the time spent in the interaction zone with a CFW present and the number of fluorescent particles (PL axons) in BLA (Fig. 5B, R^2^ = 0.5560, p = 0.0014). These correlations suggest that the lower the density of, and the higher the level of fragmentation of PL axons in BLA, the lower the amount of social interaction as assayed by OFSI. Examination of the correlations between social interaction in OFSI and PL innervation in NAc resulted in similar but slightly weaker trends to those described for BLA (Fig. 5C, D).

## Discussion

Overall we have determined that 5 day eaCSDS is sufficient to drive significant changes in both social behavior and in PL innervation of downstream structures in males and females. While defeated males display socially avoidant behavior in OFSI at multiple time points through development, defeated females show no changes in either social interaction ratio or time spent interacting at any time point. However, social testing in HCSI with freely behaving mice reveals that defeated females show aberrantly increased social behavior, including less distance between mice and more bodily contact and approach towards the other mouse by the defeated female. Both defeated males and females show decreased integrated density and mean fluorescent intensity and increased fragmentation of PL axons in BLA. Defeated males also show increased fragmentation of PL axons in NAc. Finally, we are able to show that social interaction strongly correlates with the level of PL innervation in BLA.

Fragmentation of PL axons, as measured by fluorescent particle number and size, appears consistently across BLA and NAc in defeated males and in BLA of defeated females. This measure of fragmentation is used in analysis of neurodegeneration where increases in particle number and decreases in particle size are related to degenerative processes in axons^34-36^. It may be that the fragmentation of PL axons is due to accelerated neurodegenerative processes as a result of the prolonged stress of defeat. Given that PL innervation of BLA should be complete by P21 [30], several days prior to the start of defeat, we believe that the decrease in innervation we see is not due to a halting or mistargeting of PL innervation. Additionally, research in humans has indicated that neurodegenerative disease is more common in those with histories of trauma [37, 38]. Another possible explanation for the decreases in axon innervation we see is phenotypic adaptation [39–41]. A loss of PL innervation may actually be a compensatory mechanism to support avoidant or increased affiliative behavior in unpredictable social interactions. Through this lens, the loss of PL axons may be due not to degeneration but to accelerated synaptic pruning that is compensated by enhanced synaptic strength of the remaining axons, allowing for more adaptive behavior in the context of potential threat.

In our analysis of both behavior and anatomical innervation, defeat appears to differentially affect males and females. Defeated males appear to be more threat-avoidant while defeated females demonstrate a more affiliative or possibly hypervigilant response in social interaction. These sex-specific changes in social behaviors are consistent with male-avoidant versus female-affiliative phenotypes seen in both the human and animal literature in response to early life adversity [42–46]. Avoidance of potential threat by males and increased affiliation by females can both be seen as adaptive responses in a social interaction in which the outcome is unknown. It may be that avoidance in males is driven by a distributed pattern of PL innervation loss in BLA and NAc while approach in defeated females is driven by a disruption of PL innervation in BLA alone and that these changes are forms of sex-specific circuit-level adaptations to threat rather than simply neurodegeneration.

### Limitations

While our viral GFP strategy labels the cytosol of neurons, it does not label synaptic release sites; this introduces the caveat that our strategy labels fibers of passage as well as terminal fields, making it difficult to determine if terminal fields for specific regions are uniquely affected or simply due to changes in the number of fibers of passage. However, previous work using a combined cytosolic RFP- synaptobrevin-GFP fusion construct to trace prefrontal fiber tracts shows that fibers of passage extend caudoventrally through the corpus callosum, dorsal striatum, dorsal thalamus, hypothalamus, and midbrain structures [47], excluding BLA and NAc. The labeled axons in BLA, NAc, and VTA are also punctate in appearance rather than continuous, wider diameter fibers, further suggesting that the ROIs analyzed do not contain fibers of passage [48, 49]. Nevertheless, future experiments should utilize a synaptophysin- or synaptobrevin-fused fluorophore that is specifically expressed at release sites to definitively determine changes in density of terminal fields with synaptic release sites. Additionally, our strategy is not cell type or projection type specific and thus we are unable to determine if there is a change in collateralization specific to one projection type or another. Future studies will incorporate intersectional labeling strategies to better understand how collaterals from PL cells that project to multiple targets are affected by eaCSDS.

We also note the discrepancies in sample size between male and female cohorts for the PL innervation analysis, with the female cohort (n = 6 defeated, 4 control females) being smaller and thus more susceptible to sampling errors. Despite the small size of the female cohort, the effect sizes for significant main effects of sex can all explain greater than 15% of the variance, indicating that there is a large effect size despite the small female sample.

### Future directions

While our current work is able to confirm a correlative link between PL innervation of BLA and social behavior, we have not yet employed functional manipulations in order to determine causality. In the future, we will use circuit-specific optogenetic manipulation of the PL-BLA circuit to determine whether increasing the activity of this circuit is sufficient to restore normative social behavior. However it remains to be seen if stimulation of a pathway with decreased anatomical innervation is sufficient to drive a normalization of social behavior in either sex. Future experiments may require the manipulation of other cell types or pathways if stimulation of the remaining PL axons in BLA is not sufficient to ameliorate defeat-induced social behavior. It is also critical to determine the timepoints at which axonal loss occurs after defeat. Future longitudinal experiments will examine PL innervation during and immediately after defeat to pinpoint the timepoint at which PL axons are lost. Of note, in our current study it is not possible to determine if axonal loss is reversible. In the future, we hope to use behavioral and pharmacological interventions to determine if axon loss is preventable at early timepoints and reversible at later timepoints.

Our current work is also unable to differentiate whether PL axonal loss is a sign of accelerated neurodegeneration or phenotypic adaptation. Future experiments will examine whether axons are lost due to accelerated pruning or increased inflammation using histological markers. To further distinguish between degeneration and adaptation, we plan to examine population activity in BLA in response to optogenetic stimulation of PL terminals as well as PL terminal activity in BLA; these experiments would allow us to determine if PL drive to BLA is simply weakened or if the remaining PL axons show an increased compensatory response. Overall, future work will attempt to distinguish between circuit level degeneration versus adaptation in order to determine whether the appropriate therapeutic target is neuroprotection or circuit rebalancing.

## Supplementary information

Supplemental material

## Data Availability

Behavioral videos and histological images can be provided upon request to E.E.H.
