# Supplementary material for: Early life social defeat in mice reduces prefrontal innervation of downstream structures in a regionally specific manner
Source: Transl Psychiatry. 2026 Jul 2;16:482. doi: 10.1038/s41398-026-04229-6 (PMC13601558; doi:10.1038/s41398-026-04229-6)
Supplement: Supplementary file 1 [file 41398_2026_4229_MOESM1_ESM.docx]

**Supplementary Information**

Supplemental table 1

Supplemental figures 1-3
**Supplemental table 1: ANOVA statistical results from comparison of PL innervation of downstream regions by sex and by defeat status.** Significant effects are highlighted in green and in bold font.

| **BLA integrated density** | **SS (Type III)** | **F (DFn, DFd)** | **P value** | **Partial eta-squared;**  **Cohen’s *f***  **(effect size)** | **95% CI of difference** |
| --- | --- | --- | --- | --- | --- |
| Interaction | 3.393E+13 | F (1, 23) = 0.3955 | P=0.5356 |  |  |
| Row Factor (sex) | 9.989E+13 | F (1, 23) = 1.164 | P=0.2918 |  |  |
| **Column Factor (defeat)** | **5.832E+14** | **F (1, 23) = 6.799** | **P=0.0157** | **0.228;**  **0.544** | **2.02E+6 to**  **1.76E+7** |
| Residual | 1.973E+15 |  |  |  |  |
| **BLA count** | **SS (Type III)** | **F (DFn, DFd)** | **P value** |  |  |
| Interaction | 91594 | F (1, 22) = 1.255 | P=0.2747 |  |  |
| **Row Factor (sex)** | **791938** | **F (1, 22) = 10.85** | **P=0.0033** | **0.330;**  **0.702** | **135 to 594** |
| **Column Factor (defeat)** | **319818** | **F (1, 22) = 4.381** | **P=0.0481** | **0.166; 0.446** | **-461 to**  **-2.13** |
| Residual | 1605986 |  |  |  |  |
| **BLA average size** | **SS (Type III)** | **F (DFn, DFd)** | **P value** |  |  |
| Interaction | 2963 | F (1, 22) = 0.7254 | P=0.4036 |  |  |
| **Row Factor (sex)** | **69472** | **F (1, 22) = 17.01** | **P=0.0004** | **0.436;**  **0.879** | **-162 to**  **-53.7** |
| **Column Factor (defeat)** | **39228** | **F (1, 22) = 9.605** | **P=0.0052** | **0.304;**  **0.661** | **26.8 to**  **135** |
| Residual | 89852 |  |  |  |  |
| **BLA percent area** | **SS (Type III)** | **F (DFn, DFd)** | **P value** |  |  |
| **Interaction** | **49.47** | **F(1,22) = 4.991** | **P =0.0360** | **0.185;**  **0.476** | **0.41 to**  **11.1** |
| Row Factor (sex) | 31.10 | F(1,22)=3.137 | P =0.0904 |  |  |
| Column Factor (defeat) | 11.47 | F(1,22) = 1.157 | P =0.2937 |  |  |
| Residual | 218.1 |  |  |  |  |
| **BLA mean intensity** | **SS (Type III)** | **F (DFn, DFd)** | **P value** |  |  |
| Interaction | 9378 | F(1,22) = 0.7871 | P =0.3846 |  |  |
| Row Factor (sex) | 7121 | F(1,22)= 0.5977 | P =0.4477 |  |  |
| **Column Factor (defeat)** | **53537** | **F(1,22) = 4.493** | **P =0.0455** | **0.170;**  **0.452** | **2.052 to**  **187.4** |
| Residual | 262117 |  |  |  |  |
|  |  |  |  |  |  |
| **NAc integrated density** | **SS (Type III)** | **F (DFn, DFd)** | **P value** |  |  |
| Interaction | 3.71287E+12 | F (1, 23) = 0.0603 | P=0.8081 |  |  |
| **Row Factor (sex)** | **2.722E+14** | **F (1, 23) = 4.427** | **P=0.0465** | **0.161;**  **0.439** | **-1.33E7**  **to**  **-1.16E5** |
| Column Factor (defeat) | 2.81027E+13 | F (1, 23) = 0.4570 | P=0.5057 |  |  |
| Residual | 1.414E+15 |  |  |  |  |
| **NAc count** | **SS (Type III)** | **F (DFn, DFd)** | **P value** |  |  |
| Interaction | 382761 | F (1, 23) = 2.511 | P=0.1267 |  |  |
| **Row Factor (sex)** | **2351359** | **F (1, 23) = 15.42** | **P=0.0007** | **0.401;**  **0.819** | **295 to**  **951** |
| Column Factor (defeat) | 369461 | F (1, 23) = 2.423 | P=0.1332 |  |  |
| Residual | 3506436 |  |  |  |  |
| **NAc average size** | **SS (Type III)** | **F (DFn, DFd)** | **P value** |  |  |
| **Interaction** | **8545** | **F (1, 23) = 12.67** | **P=0.0017** | **0.355;**  **0.742** | **31.4 to**  **119** |
| Row Factor (sex) | 553.9 | F (1, 23) = 0.8211 | P=0.3742 |  |  |
| Column Factor (defeat) | 12.99 | F (1, 23) = 0.0193 | P=0.8909 |  |  |
| Residual | 15516 |  |  |  |  |
| **NAc percent area** | **SS (Type III)** | **F (DFn, DFd)** | **P value** |  |  |
| Interaction | 8.373 | F(1,23) =1.012 | P=0.3248 |  |  |
| Row Factor (sex) | 8.589 | F(1,23)=1.038 | P=0.3188 |  |  |
| Column Factor (defeat) | 1.212 | F(1,23) =0.1465 | P=0.7054 |  |  |
| Residual | 190.2 |  |  |  |  |
| **NAc mean intensity** | **SS (Type III)** | **F (DFn, DFd)** | **P value** |  |  |
| Interaction | 3808 | F(1,23) = 0.4160 | P=0.5253 |  |  |
| **Row Factor (sex)** | **56993** | **F(1,23)=6.225** | **P=0.0202** | **0.213;**  **0.520** | **-177 to**  **-16.6** |
| Column Factor (defeat) | 2385 | F(1,23) =0.2605 | P=0.6146 |  |  |
| Residual | 210562 |  |  |  |  |
|  |  |  |  |  |  |
| **VTA integrated density** | **SS (Type III)** | **F (DFn, DFd)** | **P value** |  |  |
| Interaction | 1.02E+14 | F (1, 22) = 1.889 | P=0.1831 |  |  |
| Row Factor | 1.87E+13 | F (1, 22) = 0.3468 | P=0.5619 |  |  |
| Column Factor | 4.58E+12 | F (1, 22) = 0.08478 | P=0.7736 |  |  |
| Residual | 1.19E+15 |  |  |  |  |
| **VTA count** | **SS (Type III)** | **F (DFn, DFd)** | **P value** |  |  |
| Interaction | 17139 | F (1, 22) = 0.2629 | P=0.6133 |  |  |
| Row Factor | 12997 | F (1, 22) = 0.1994 | P=0.6596 |  |  |
| Column Factor | 80482 | F (1, 22) = 1.234 | P=0.2785 |  |  |
| Residual | 1434308 |  |  |  |  |
| **VTA average size** | **SS (Type III)** | **F (DFn, DFd)** | **P value** |  |  |
| Interaction | 55.83 | F (1, 22) = 0.4364 | P=0.5157 |  |  |
| Row Factor | 12.8 | F (1, 22) = 0.1001 | P=0.7547 |  |  |
| Column Factor | 3.008 | F (1, 22) = 0.02351 | P=0.8795 |  |  |
| Residual | 2814 |  |  |  |  |
| **VTA percent area** | **SS (Type III)** | **F (DFn, DFd)** | **P value** |  |  |
| Interaction | 6.048 | F(1,22) =0.2987 | P=0.5902 |  |  |
| Row Factor (sex) | 14.71 | F(1,22)=0.7263 | P=0.4033 |  |  |
| Column Factor (defeat) | 0.076 | F(1,22) =0.0008 | P=0.9778 |  |  |
| Residual | 445 |  |  |  |  |
| **VTA mean intensity** | **SS (Type III)** | **F (DFn, DFd)** | **P value** |  |  |
| Interaction | 6867 | F(1,22) =0.4239 | P=0.5218 |  |  |
| **Row Factor (sex)** | **79381** | **F(1,22)=4.900** | **P=0.0375** | **0.182;**  **0.403** | **7.279 to**  **223.5** |
| **Column Factor (defeat)** | 4391 | F(1,22) =0.2711 | P=0.6078 |  |  |
| Residual | 356426 |  |  |  |  |

**Supplemental figure 1: PL injection sites show no difference between males and females or control and defeated groups.** A) PL injection site area (averaged across 5 sections) in control (n = 7 males (blue), 4 females (pink)) and defeated (n = 10 males, 6 females) animals. B) Injection site integrated density (averaged across 5 sections). C) BLA integrated density as a function of injection site area.

**Supplemental figure 2: Percent area and mean fluorescent intensity in BLA and NAc using 300 um ROI with default thresholding.** A) Percent area of PL axons in BLA (interaction between sex and defeat, 2 way ANOVA, F(1,22) = 4.991, p = 0.0360, Tukey HSD for control vs. defeated males, p = 0.0288). B) Mean fluorescent intensity of PL axons in BLA (main effect of sex, 2 way ANOVA, F(1,22) = 4.493, p = 0.0455). C) Percent area of PL axons in NAc. D) Mean fluorescent intensity of PL axons in NAc (main effect of sex, 2 way ANOVA, F(1,23) = 6.225, p = 0.0202).

**Supplemental figure 3: PL innervation of VTA is not significantly affected by defeat or sex.** A) Integrated density of PL axons in VTA (males: n = 7 control, 10 defeated animals; females: n = 4 control, 6 defeated animals). B) Percent area occupied by PL axons in VTA (males: n = 7 control, 9 defeated animals; females: n = 4 control, 6 defeated animals). C) Mean fluorescent intensity (main effect of sex, 2 way ANOVA, F(1,22)=4.900, p = 0.0375, 95%CI: males [698.3, 874.3], females [645.1, 705.7]). D) Number (count) of fluorescently labeled PL particles in VTA (males: n = 7 control, 9 defeated animals; females: n = 4 control, 6 defeated animals). E) Average size of fluorescently labeled particles in BLA (males: n = 7 control, 9 defeated animals; females: n = 4 control, 6 defeated animals). F) Integrated density of PL axons in NAc (males: n = 7 control, 10 defeated animals; females: n = 4 control, 6 defeated animals). G) Number (count) of fluorescently labeled particles in NAc (males: n = 7 control, 10 defeated animals; females: n = 4 control, 6 defeated animals) (males: unpaired one-tailed t test, p = 0.0365).
